# Step by Step: Investigating Children’s Physical Activity and Enjoyment in Outdoor Walking with Their Parents

**DOI:** 10.3390/healthcare13141721

**Published:** 2025-07-17

**Authors:** Patrick M. Filanowski, Jeremy A. Steeves, Emily Slade

**Affiliations:** 1 Department of Sport Science & Management, Xavier University, Cincinnati, OH 45207, USA; steevesj@xavier.edu; 2 Department of Biostatistics, University of Kentucky, Lexington, KY 40506, USA

**Keywords:** MVPA, parent–child, family, accelerometer

## Abstract

**Background/Objectives**: Although public health organizations encourage family walking, no studies have examined children’s physical activity and enjoyment during outdoor parent–child walks. This study addresses those gaps by examining children’s moderate-to-vigorous physical activity (MVPA) and enjoyment during outdoor walks with their parents, along with parental barriers and their relationship with parent’s self-efficacy and co-activity minutes. **Methods**: Fifty parent–child dyads (children aged 6–12 years) completed 10 min, self-paced outdoor walks while wearing waist-worn ActiGraph monitors. Parents reported perceived barriers to walking outdoors with their child and self-efficacy for supporting their child’s daily physical activity. **Results**: Children reported high enjoyment (mean = 5.1 on a six-point scale) and attained high physical activity intensity (71.3% of time in MVPA, 22.0% in vigorous activity, mean step count = 1200). Parents reported an average of 2.6 barriers (SD = 1.0) to walking outdoors with their child, with poor weather (70%) and lack of time (70%) reported most frequently. Each additional barrier was associated with a 1.3-point reduction in parents’ self-efficacy (*p* = 0.007). Two barriers (‘diverse interests between parent and child’ and ‘other parent-suggested barriers’) were significantly associated with fewer co-activity minutes per week (*p* < 0.001). **Conclusions**: Our study highlights the benefits of parent–child outdoor walking for promoting MVPA and enjoyment in children. Because perceived barriers may lower parents’ self-efficacy in supporting their child’s physical activity, addressing these barriers may be essential for the success of family-based interventions that encourage walking together outdoors.

## 1. Introduction

Less than a third of children in the United States participate in the recommended 60 min of daily moderate-to-vigorous physical activity (MVPA) [1]. Because of these low levels of physical activity, promoting children’s physical activity participation has become an important public health priority [2]. Regular participation in physical activity has been positively associated with several health benefits for children including improved cardiorespiratory endurance, muscular fitness, weight status, bone health, and cardiometabolic health [3]. The formative years before adolescence may be a critical time to develop healthy physical activity habits in children, due to the declining levels of physical activity and increasing levels of sedentary behavior frequently seen during adolescence [4,5]. While there are numerous outlets where children can be physically active (e.g., organized sport, active transportation, school), co-participation in physical activity with their parents may be a unique and equitable opportunity to promote the enhancement and adherence of children’s physical activity levels [6,7].

The United States Report Card on Physical Activity for Children and Youth, published biennially by the Physical Activity Alliance, assesses several indicators that inform policies and initiatives to improve children’s physical activity levels [1]. Since 2014, the “Family and Peers” category has been graded as “Incomplete” due to insufficient or inadequate information available to assign an A–F letter grade, stressing the need for research on how families and peers influence children’s physical activity. Specifically, the Physical Activity Alliance recommends examining parents’ abilities and confidence to influence their children to be more physically active and opportunities for parents to engage in physical activity with their children [1]. Parents’ self-efficacy to influence their child’s behaviors can positively impact their child’s involvement in physical activity [8]. Bruijns et al. [9] found that parents of young children who participate in movement-based activities have reported high self-efficacy for facilitating physical activity opportunities and moderate self-efficacy in serving as a positive role model for their children’s physical activity. When parents feel confident about engaging in and modeling healthy behaviors with their children, such as physical activity, it may increase the likelihood that children will adopt similar healthy habits.

Parents play an important role in their children’s physical activity engagement by providing encouragement, resources, and co-participating in enjoyable physical activities together [3]. Public health initiatives that encourage parent–child co-participation in physical activity may be an effective strategy to increase levels of physical activity in children [1,10,11]. Including parents in their children’s nutrition and physical activity interventions has produced beneficial results in the prevention and treatment of childhood overweight and obesity [12]. Parent–child co-participation in physical activity has been found to promote parent–child enjoyment of physical activity participation, which can contribute to the engagement of MVPA in both children and adults [13,14]. Parents’ and their children’s levels of physical activity participation are often related, but the extent of this connection can vary across different contexts [15,16]. Also, it can be challenging for parents and children to find specific physical activities that they both enjoy and result in MVPA [13].

Several public health organizations including the Centers for Disease Control and Prevention [17], the American Heart Association [18], and the Office of Disease Prevention and Health Promotion [19] promote walking, the most popular form of physical activity [20], as an activity that parents and children can participate in together. The 2018 Physical Activity Guidelines for Americans specifically recommends walking as an activity for adults and children to meet the federal recommendations for MVPA [3]. Co-participation in walking can be easily incorporated into families’ daily schedules, increasing physical activity levels in children and parents [21,22]. Brisk walking in an indoor research laboratory setting was found to be an acceptable physical activity for co-participation in parent–child dyads when compared to other physical activities such as jumping games, body-weight exercises, dancing, and tag games [13]. A more recent study showed that parents who participated in outside walking sessions with their children attained high percentages of time spent in MVPA (96.6%), and enjoyed walking more with their child than walking alone [23]. However, to our knowledge, no studies have assessed MVPA and enjoyment of children during co-participation in walking with their parents outside of a research laboratory setting.

Despite widespread recommendations encouraging parents and children to walk together, there is a lack of research measuring children’s MVPA and enjoyment during parent–child walking. Existing studies have examined parent and child activity levels separately or focused on co-participation in indoor, laboratory-based settings [13,23]. To our knowledge, no studies have assessed how much MVPA children accumulate, or how much they enjoy walking, during outdoor walks with their parents. This represents a critical gap in the literature, especially given the accessibility and public health messaging promoting walking as a family-based physical activity.

The purpose of the present study was to investigate children’s MVPA levels and enjoyment of walking with their parents during self-paced, outdoor walking sessions and to examine the barriers to co-participation in outdoor walking at home. The first aim was to estimate the proportion of children’s time spent in MVPA while walking outdoors with their parents. The second aim was to assess whether children enjoy walking outdoors with their parents during co-participation. Finally, recognizing that walking is often the most popular physical activity for families promoted for parents and children to participate in jointly [17,18,19], the third aim was to identify parents’ perceived barriers to walking outdoors together with their child in their home environment and examine how these barriers are related to the following: (1) parents’ self-efficacy for helping their child attain the recommended levels of physical activity and (2) the actual minutes per week that parents and children spend being physically active together.

## 2. Materials and Methods

Fifty parent–child dyads, comprising one adult (34–50 years old) and one child per dyad (6–12 years old), participated in the study via private sessions on the campus of Xavier University in Cincinnati, OH, USA. Each session consisted of obtaining consent and assent from participants, measuring height and weight, completing 10 min self-paced outdoor walking sessions for each dyad, and administering questionnaires for parents to report potential barriers to walking with their child and self-efficacy for helping their child attain recommended levels of physical activity. The Institutional Review Board of Xavier University reviewed and approved the study (Protocol #20-075).

All 10 min outdoor walking sessions began at the same location, and participants were directed to walk within a specific area. As the first study to investigate shared outdoor walking in parents and children, we chose a manageable timeframe that would be acceptable across dyads and minimize participant burden in a field-based setting. We selected 10 min walking bouts because they offered a feasible and realistic duration that aligns with public health guidelines, which emphasize that any duration of physical activity contributes to the health benefits associated with accumulated physical activity volume [3].

A researcher-controlled walking protocol was chosen to ensure consistency in the walking environment, route, duration, and procedures across all dyads, supporting comparability of the data collected. Participants were instructed to choose a walking pace that was comfortable yet provided good exercise, with the flexibility to adjust speed, pace, and effort level throughout the walk [23]. Children’s enjoyment levels of walking were assessed immediately after each bout using a six-point Likert scale ranging from “1—Do not like it at all” to “6—Like it very much”. Physical activity intensity and step counts were assessed using ActiGraph wGT3X-BT (Pensacola, FL, USA) right hip-worn accelerometers (raw data collected at 30 Hz sampling rate) and processed using ActiLife version 6.13.4. Physical activity intensity levels for the child participants were determined using fifteen-second epoch lengths and the Evenson cut points [24,25].

From a list of ten potential barriers (not enough time, lack of available places to walk, neighborhood is not safe enough, diverse interests between you and your child, tiredness/no motivation to walk outdoors, poor weather, would rather participate in physical activities with your child other than walking, health-related concerns, no barriers, and ‘other barriers’), parents were asked to identify which would be barriers to walking with their child outdoors. For the ‘other barriers’ option, parents were invited to describe these barriers through an open-ended response.

To assess parents’ self-efficacy for helping their child attain recommended levels of physical activity, parents were asked to indicate how confident they were that they could help their child get one hour of moderate intensity physical activity every day in the following four scenarios: (1) when there are too many other things to worry about, (2) when money is tight, (3) when you do not like to exercise or play, and (4) when your child is tired. Each scenario had answer options ranging from 1 to 5, where 1 represented “not sure” and 5 represented “extremely sure.” Parents’ overall self-efficacy for helping their child attain recommended levels of physical activity was calculated as a composite score (sum) of these four items [26]. Parents also reported the actual minutes per week that they are physically active with their child. Currently, there is no validated self-report instrument designed to assess shared physical activity between parents and children. As such, we asked parents to directly report the number of minutes per week they were physically active with their child, providing a practical and contextually relevant estimate of shared activity.

Children’s enjoyment ratings and accelerometer-measured physical activity (percentage of time in MVPA, percentage of time in vigorous physical activity, step counts) were summarized with mean and standard deviation. It was hypothesized that children would have high levels of enjoyment and attain most of the time in MVPA (as opposed to sedentary behavior or light physical activity) during the 10 min outdoor walking session with their parent. This hypothesis was informed by previous research indicating that children’s enjoyment of physical activity can influence their MVPA and children can acquire MVPA when walking with a parent indoors [13,14]. It was also hypothesized that parents who indicated having more barriers to walking outdoors with their child would have: (1) lower self-efficacy for helping their child attain recommended levels of physical activity, and (2) lower minutes per week that they were physically active with their child. These hypotheses were informed by previous research showing that parents often face various barriers, such as time constraints, children’s preferences, and environmental barriers, which can affect their ability to support their child’s physical activity [27]. To test (1), a linear regression model was fit relating the total number of personal barriers to the self-efficacy score for each child’s parent. To explore whether there are individual barriers that are more or less impactful than others, separate linear regression models were used to examine the association between each individual personal barrier (yes/no) with the self-efficacy score. Using a similar approach for (2), a Poisson regression model was used to account for the zero-bounded and right-skewed distribution of the minutes per week that parents were physically active with their child. The robust sandwich variance estimator was used to account for overdispersion. Primary analyses were performed without covariate adjustment due to the focus on identifying associations rather than estimating causal effects and concerns about overfitting given the modest sample size. Secondary analyses were performed including adjustment for child body mass index (BMI), parent education level, and family income; these analyses are exploratory and should be interpreted with caution due to the possibility of overfitting. For all analyses, findings were considered statistically significant when *p* < 0.05. Analyses were performed in R version 4.2.0 [28].

## 3. Results

Complete demographic and anthropometric characteristics of study participants can be found in Table 1. Child participants ranged in age from 6 to 12 with a mean age of 9.3 years old (SD = 1.9). There were slightly more female children (54.0%) in the study. Child participants were predominantly White (76.0%) and from households with an annual income greater than $100,000 (58.0%). Most children were normal weight or underweight (62.0%); 16.0% were overweight, and 22.0% were obese.

Children attained high levels of enjoyment while walking outdoors with their parent during the study, characterized by a mean enjoyment rating of 5.1 (SD = 1.0) on a six-point scale (Table 2). Children also attained high levels of physical activity while walking with their parent: on average, children spent 71.3% of the session in MVPA, 22.0% of the session in vigorous physical activity, and took 1200 steps during the ten-minute outdoor walking session (Table 2).

**Figure 1:**
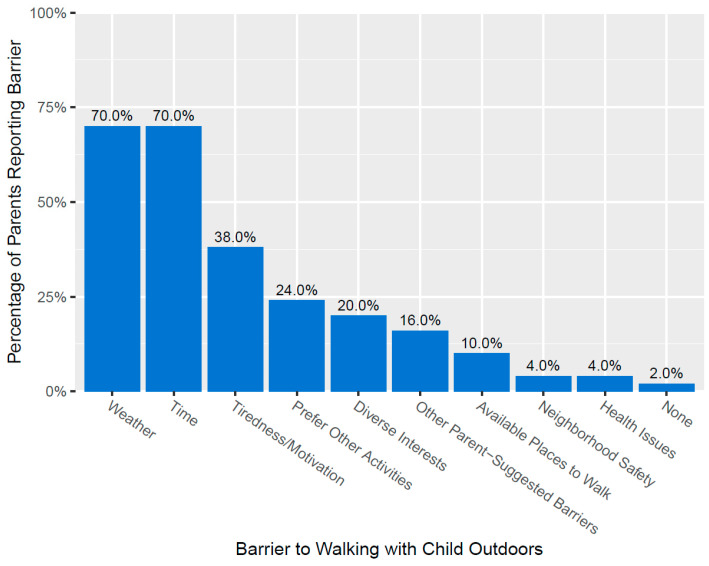
Percentage of parents reporting each barrier to walking with their child outdoors. “Prefer other activities” refers to ‘would rather participate in physical activities with your child other than walking.’ “Diverse interests” refers to ‘diverse interests between you and your child.’ “Other parent-suggested barriers” included: child’s lack of interest in walking outdoors (n = 4, 8.0%), other children (n = 2, 4.0%), child tiring out too quickly (n = 1, 2.0%), and mindset (n = 1, 2.0%).

Overall, parents reported having up to five different barriers to walking outdoors with their child, with the mean number of barriers per parent being 2.6 (SD = 1.0). The most frequent barriers parents reported to walking outdoors with their child were “poor weather” (n = 35, 70.0%) and “not enough time” (n = 35, 70.0%) (Figure 1). “Tiredness/no motivation to walk outdoors”, “would rather participate in physical activities with your child other than walking”, and “diverse interests between you and your child” were also reported as barriers by 38.0%, 24.0%, and 20.0% of parents, respectively (Figure 1). Sixteen percent of parents reported that they had other barriers that were not listed, including the following parent-suggested barriers: “child’s lack of interest in walking outdoors” (n = 4, 8.0%), “other children” (n = 2, 4.0%), “child tiring out too quickly” (n = 1, 2.0%), and “mindset” (n = 1, 2.0%).

When considering the total number of barriers to walking outdoors with their child, each additional barrier that a parent indicated having was associated with a 1.26-point decrease in the parents’ composite self-efficacy score for helping their child attain at least one hour of moderate intensity physical activity every day (*p* = 0.007, Table 3). In other words, as parents face more barriers, they tend to feel less confident in their ability to support their child’s daily physical activity. This association remained significant after adjustment for child BMI, parent education level, and family income (Table S1). When each individual barrier was considered, the parents’ self-efficacy for helping their child attain at least one hour of moderate intensity physical activity every day was significantly lower amongst those who indicated they had diverse interests to their child (*p* = 0.024) or other parent-suggested barriers (*p* = 0.039) (Table 3). Other parent-suggested barriers remained significantly associated with parents’ self-efficacy after adjustment for child BMI, parent education level, and family income (Table S1).

The median number of minutes per week that parents indicated being active with their child was 60.0 min (IQR: 30.0, 90.0) (Table 1). When considering the total number of barriers to walking with their child, each additional barrier a parent reported having was associated with a 19.1% decrease in the minutes per week that the parent and child were physically active together, though this association was not statistically significant (*p* = 0.062, Table 4). For a parent reporting the mean of 3 barriers, that corresponds to an approximate 27.0 min reduction from the median of 60.0 min per week, indicating a meaningful, though not statistically significant, reduction in shared physical activity time. When each individual barrier was considered, the minutes per week spent being active with the child were significantly lower among those who reported having diverse interests from their child (*p* < 0.001) or other parent-suggested barriers (*p* < 0.001) (Table 4). Specifically, parents who identified diverse interests as a barrier spent 57.1% fewer minutes being active with their child (an approximate 34.3 min reduction from the median 60.0 min per week) compared to those who did not report that barrier, after adjusting for other possible barriers (Table 4). Parents who reported other parent-suggested barriers spent 78.7% fewer minutes per week in shared physical activity with their child (an approximate 47.2 min reduction from the median 60.0 min per week) compared to those who did not report such barriers, after adjusting for other possible barriers (Table 4). These associations remained significant after adjusting for child BMI, parent education level, and family income (Table S2).

## 4. Discussion

The present study investigated children’s MVPA levels and enjoyment during self-paced, outdoor walking sessions with their parents. Additionally, the study explored parent-perceived barriers to co-participation in outdoor walking at home, including examining the relationship between parent-perceived barriers and two outcomes: (1) parents’ self-efficacy for helping their child attain the recommended levels of physical activity, and (2) minutes per week that the child and parent are physically active together. Children achieved a substantial amount of time in MVPA during the outdoor walking sessions with their parents and exhibited high levels of enjoyment. Parents’ most prevalent barriers to walking with their children outdoors were weather conditions and time constraints. A higher number of identified barriers was associated with lower average parental self-efficacy regarding their capacity to support their child in achieving recommended levels of physical activity. Two barriers, ‘diverse interests between the parent and child’ and ‘other parent-suggested barriers’, were significantly associated with a lower amount of time per week that the child and parent are physically active together.

Significant health benefits are associated with regular MVPA in children, including improvements in cardiovascular health, better weight management, and enhanced mental well-being [3]. Positive parental support and encouragement play pivotal roles in shaping a child’s perception of physical activity as a fun and enjoyable experience [29]. Furthermore, the involvement of parents with their children during indoor laboratory physical activities has been associated with increased MVPA and enjoyment in children [13]. We built on earlier research examining parent–child walking indoors [13] by assessing shared parent–child walking in an outdoor environment. Consistent with prior findings on indoor, laboratory-based walking [13], our results demonstrate that shared parent–child walking outdoors can elicit similarly high levels of enjoyment and MVPA, reinforcing the robustness of walking as an acceptable activity across diverse environments. Other research has highlighted high MVPA and enjoyment in parents during shared outdoor walking with their children [23]. Our study adds new evidence by demonstrating that children can also achieve high levels of MVPA and enjoyment, an aspect not previously examined.

The barriers to walking with their children outdoors that were reported by parents in our study are consistent with self-reported barriers to walking identified in other studies [30,31]. Shyleyko et al. [31] evaluated children’s physical activity using caregiver-administered surveys and found, as in our study, motivation and time constraints were commonly reported barriers, while access to physical activity spaces was not a barrier to being active. Poor weather, time, and lack of motivation emerged as the top three barriers to walking in adults participating in a community-based walking program [30]. Although the adults in the aforementioned study did not exclusively comprise parents, they shared the same top three barriers to walking as parents in our study. Notably, a larger percentage of parents in our study reported having these barriers compared to the participants in Richards & Woodcox [30]. Compared to adults walking alone, parents who walk with their children may encounter additional challenges that need to be addressed to engage and maintain physical activity behaviors. Within the context of promoting children’s health behaviors, parents experience more barriers to supporting their child’s physical activity compared to other health behaviors, including screen time reduction, healthy eating, and sleep [27]. Although our walking protocol was conducted in a researcher-controlled outdoor setting, the reported barriers, such as lack of time, poor weather, and low motivation, are challenges that are relevant to co-participation in home and community environments.

Families may better manage time barriers by intentionally scheduling shared physical activity as part of their daily or weekly routines [18,19]. Breaking physical activity into shorter bouts throughout the day, such as multiple 10 min walks, may also help overcome perceived time constraints while still accumulating meaningful physical activity. To address weather-related barriers, families can plan for inclement conditions by identifying accessible indoor alternatives, such as walking at local malls, or preparing appropriate clothing and gear to remain comfortable outdoors in less favorable weather. These practical strategies can help families overcome common barriers and support consistent parent–child co-activity. In addition to time, poor weather, and low motivation, a notable proportion of parents in our study reported preferring other activities as a barrier to walking with their child. This barrier may reflect instances where either the parent, the child, or both have different interests or prioritize alternative forms of physical activity. Divergent activity preferences between parents and children could make shared walking less appealing, highlighting the importance of discovering flexible and varied co-activity options that align with family interests. Future studies might consider strategies to identify other shared activities that are enjoyable for both parents and children and use them to encourage increased opportunities for shared physical activity participation. These findings can help inform future interventions by highlighting the importance of offering flexible, family-centered approaches that account for diverse interests and time constraints. Findings in our study reinforce shared outdoor walking as a promising approach for parent–child physical activity interventions, while also emphasizing to the need to develop strategies that address these barriers in real-world settings.

Previous research supports that both parent-reported barriers and parental self-efficacy to influence their child’s physical activity are significant determinants of children’s physical activity levels independent of family income [32]. We found that the number of reported barriers was negatively associated with parental self-efficacy, emphasizing the substantial impact of identified barriers on parental confidence in supporting recommended levels of their child’s physical activity. In another study, kindergarten children of parents with lower self-efficacy for promoting physical activity had significantly less daily physical activity than children of parents with higher self-efficacy for promoting physical activity [33]. It is important to consider the impact that both higher parent-perceived barriers and lower parental self-efficacy could have as detriments toward parents’ ability to support their children’s physical activity levels. In addition, certain individual barriers were found to be more impactful than others. Diverse interests between parents and children and other parent-suggested barriers, including the child’s lack of interest, were significantly associated with lower self-efficacy in parents. Other studies have identified that these diverse interests and activity preferences can also affect participation in shared physical activities between parents and children [34]. For instance, when parents and children have conflicting preferences for shared physical activities, it may create a challenge for parents in promoting joint activities. While walking is the most frequently reported activity in adults [20] and contributes significantly to children’s overall physical activity [35], children may prefer other activities such as high-energy, engaging activities or sedentary activities [36,37]. These differing preferences can potentially lead to a belief that it is difficult to find an activity both parents and children can participate in together or enjoy, further reducing the likelihood that physical activity will be a feasible family behavior. Since parents strongly influence their children’s MVPA behaviors, interventions that target the entire family present an opportunity to improve physical activity habits [38]. Additional research is needed to foster shared physical activity through family-centered interventions and planning strategies to bridge these potential preference gaps.

Our study had several strengths worth highlighting. Although the study took place in a controlled setting, it was the first to assess children’s MVPA and enjoyment levels during outdoor co-participation in walking with their parents, rather than in an indoor laboratory environment, while also examining barriers to walking. Our study, which took a didactic approach to examining both children and parents, addresses a gap identified in the 2024 U.S. Report Card on Physical Activity for Children and Youth regarding the role of families in promoting children’s physical activity behaviors [1]. Future studies should assess children’s MVPA and enjoyment while walking with multiple caregivers and/or multiple children, which may affect both physical activity and enjoyment levels. Second, we used a variety of methodological approaches in collecting our data, including using objective ActiGraph wGT3X-BT activity monitors to assess physical activity, an easy-to-implement Likert scale for enjoyment, and subjective questionnaires to assess self-efficacy and barriers, which enhanced the comprehensiveness of our findings. We also analyzed both total barriers and the impact of individual barriers, which offered nuanced insights into how various barriers may influence parent–child activity levels and parental self-efficacy for walking with their children.

Our study also had some limitations. All participants completed our study during the summer months in Cincinnati, OH, USA, often during mostly fair-weather conditions, which may differ from what other populations may experience during outdoor walking. The location of our study was on sidewalk paths of a park-like university campus, which may not be like the settings that parents and children have access to in their communities. While we recruited a relatively balanced number of male and female children, our sample mostly comprised normal-weight children who are White and from higher-income families, which may limit the generalizability of our findings when comparing to other racial and socioeconomic groups. This demographic homogeneity, lower obesity prevalence in children, and safe campus environment may limit the generalizability of our findings, which could influence perceptions of barriers such as safety and time for physical activity. Primary regression analyses did not include covariate adjustment for demographic or socioeconomic variables, which may influence both perceived barriers and physical activity. While preliminary adjusted models yielded similar findings, future research with larger samples may benefit from fully accounting for these potential confounders. Additionally, because no validated self-report instrument exists for assessing shared physical activity between parents and children, we relied on parent-reported estimates of minutes per week of shared physical activity, which may be subject to reporting bias. Lastly, the cross-sectional design of our study prevents examination of how the relationship between parents’ perceived barriers to walking with their children outdoors and children’s physical activity behaviors may evolve over time. More research is needed to assess the effects of parental barriers and self-efficacy for helping their children meet physical activity recommendations throughout different stages of their childhood.

## 5. Conclusions

Our study explored parent–child co-participation in outdoor walking and identified key barriers parents have to walking with their children. Given the limited data on how families and peers influence children’s physical activity, our findings offer novel insights. Walking together emerged as a promising way for children to achieve health-enhancing MVPA while providing meaningful, enjoyable interactions with their parents. However, several barriers, including weather, time constraints, tiredness/motivation, and preference for other activities were observed, which may be associated with decreased physical activity participation in some parents and children. Barriers were found to be negatively associated with parents’ self-efficacy in supporting their child’s physical activity, with specific barriers of diverse interests and lack of shared preferences being particularly significant. Our results highlight the need for more research to explore the impact of targeted interventions that address both parental and child preferences for certain physical activities, as well as strategies to mitigate common barriers to walking.

## Figures and Tables

**Table 1:** Baseline characteristics of study participants.

Variable	Parent–Child Dyads (n = 50)
Child’s age	9.3 ± 1.9
Child’s gender identity	
Female	27 (54.0%)
Male	23 (46.0%)
Child’s race	
Asian	1 (2.0%)
Black or African American	7 (14.0%)
Mixed race	2 (4.0%)
Native Hawaiian or other Pacific Islander	1 (2.0%)
White	38 (76.0%)
Prefer not to respond	1 (2.0%)
Child’s body mass index (BMI)	
Normal weight or underweight	31 (62.0%)
Overweight	8 (16.0%)
Obese	11 (22.0%)
Annual household income	
$60,000 or under	6 (12.0%)
$60,001–$80,000	6 (12.0%)
$80,001–$100,000	6 (12.0%)
$100,001 or over	29 (58.0%)
Prefer not to respond	3 (6.0%)
Parents’ self-efficacy for helping their child be physically active	
When there are too many other things to worry about	2.5 ± 1.2
When money is tight	3.6 ± 1.2
When you do not like to exercise or play	2.8 ± 1.2
When your child is tired	1.9 ± 0.8
Composite score (sum of four items above)	10.9 ± 3.4
Minutes per week that child and parent are physically active together	60.0 [IQR: 30.0, 90.0]

Continuous variables are summarized as mean ± standard deviation for variables that are approximately normally distributed or summarized as median [first quartile, third quartile] otherwise. Categorical variables are summarized as n (%). Parents’ self-efficacy for helping their child be physically active more specifically refers to their self-efficacy for helping their child attain at least one hour of moderate intensity physical activity every day under various scenarios. The first four self-efficacy items are measured on a scale from 1 to 5 where 1 = not sure and 5 = extremely sure, and the composite self-efficacy score is the sum of the four individual self-efficacy items. Abbreviations: BMI, body mass index; IQR, interquartile range.

**Table 2:** Children’s enjoyment and physical activity during ten-minute outdoor walking sessions.

	Overall (n = 50)	Normal Weight or Underweight (n = 31)	Overweight (n = 8)	Obese (n = 11)
*Children’s Enjoyment*				
Enjoyment scale (1–6)	5.1 ± 1.0	5.1 ± 1.1	5.3 ± 0.5	5.2 ± 1.0
*Children’s Physical Activity*				
Percent time spent in MVPA	71.3% ± 37.7%	71.2% ± 37.1%	87.8% ± 32.5%	59.8% ± 41.5%
Percent time spent in vigorous PA	22.0% ± 34.2%	19.9% ± 33.7%	49.4% ± 39.5%	7.7% ± 20.0%
Step counts	1200 ± 118.8	1242 ± 92.8	1208 ± 105.1	1079 ± 117.6

Summaries are reported as mean ± standard deviation for all children in the sample (n = 50) and for subgroups defined by the child’s body mass index (normal weight or underweight (n = 31), overweight (n = 8), obese (n = 11)). Enjoyment is measured using a six-point Likert scale ranging from “1—Do not like it at all” to “6—Like it very much”. Abbreviations: MVPA, moderate-to-vigorous physical activity; PA, physical activity.

**Table 3:** Associations between total and individual barriers and parents’ self-efficacy for helping their child attain recommended levels of physical activity.

	Beta Coefficient (95% CI)	*p*-Value
*Model 1: Number of barriers*		
Number of barriers	−1.263 (−2.159, −0.367)	0.007 *
*Model 2: Individual barriers*		
Weather	0.058 (−2.018, 2.135)	0.955
Time	−0.974 (−3.148, 1.120)	0.371
Tiredness/Motivation	−0.774 (−2.789, 1.242)	0.443
Prefer Other Activities	−0.377 (−2.635, 1.881)	0.738
Diverse Interests	−2.732 (−5.080, −0.385)	0.024 *
Available Places to Walk	−0.368 (−4.304, 3.568)	0.851
Neighborhood Safety	−5.474 (−11.527, 0.580)	0.075
Other Parent−Suggested Barriers	−2.821 (−5.490, −0.152)	0.039 *

Results are from two separate linear regression models with outcome of parents’ composite self-efficacy score for helping their child attain at least one hour of moderate intensity physical activity every day (scored from 4 to 20 where higher numbers indicate higher self-efficacy). “Prefer other activities” refers to ‘would rather participate in physical activities with your child other than walking.’ “Diverse interests” refers to ‘diverse interests between you and your child.’ “Other parent-suggested barriers” included: child’s lack of interest in walking outdoors (n = 4, 8.0%), other children (n = 2, 4.0%), child tiring out too quickly (n = 1, 2.0%), and mindset (n = 1, 2.0%). * indicates *p* < 0.05.

**Table 4:** Poisson regression results for examining associations with minutes per week that the child is physically active with their parent.

	Rate Ratio (95% CI)	*p*-Value
*Model 1: Number of barriers*		
Number of barriers	0.819 (0.664, 1.010)	0.062
*Model 2: Individual barriers*		
Weather	0.998 (0.645, 1.544)	0.991
Time	0.935 (0.633, 1.382)	0.736
Tiredness/Motivation	0.913 (0.500, 1.667)	0.766
Prefer Other Activities	1.162 (0.725, 1.861)	0.533
Diverse Interests	0.429 (0.272, 0.678)	<0.001 *
Available Places to Walk	0.542 (0.206, 1.429)	0.216
Neighborhood Safety	0.596 (0.151, 2.356)	0.461
Other Parent-Suggested Barriers	0.213 (0.104, 0.437)	<0.001 *

Results are from two separate Poisson regression models with the outcome of minutes per week that the child is physically active with their parent. Rate ratios are exponentiated coefficients from the Poisson regression model, and the robust sandwich variance estimator was used to calculate confidence intervals and p-values. “Prefer other activities” refers to ‘would rather participate in physical activities with your child other than walking.’ “Diverse interests” refers to ‘diverse interests between you and your child.’ “Other parent-suggested barriers” included: child’s lack of interest in walking outdoors (n = 4, 8.0%), other children (n = 2, 4.0%), child tiring out too quickly (n = 1, 2.0%), and mindset (n = 1, 2.0%). * indicates *p* < 0.05.

## Data Availability

Data available upon reasonable request.

## Supplementary Materials

The following supporting information can be downloaded at: https://www.mdpi.com/article/10.3390/healthcare13141721/s1, Table S1. Associations between total and individual barriers and parents’ self-efficacy for helping their child attain recommended levels of physical activity, after adjusting for child body mass index, parent education level, and family income. Table S2. Poisson regression results for examining associations with minutes per week that the child is physically active with their parent, after adjusting for child body mass index, parent education level, and family income.
