# Supplementary material for: Step by Step: Investigating Children’s Physical Activity and Enjoyment in Outdoor Walking with Their Parents
Source: Healthcare (Basel). 2025 Jul 17;13(14):1721. doi: 10.3390/healthcare13141721 (PMC12294517; doi:10.3390/healthcare13141721)
Supplement: Supplementary file 1 [file healthcare-13-01721-s001.zip › healthcare-3730986-supplementary.pdf]

Supplemental Table S1. Associations between total and individual barriers and parents' self-efficacy for helping their child attain recommended levels of physical activity, after adjusting for child body mass index, parent education level, and family income.

	Beta Coefficient (95% CI)	P-Value
Model 1: Number of barriers		
Number of barriers	-0.986 (-1.914, -0.057)	0.038*
Model 2: Individual barriers		
Weather	0.883 (-1.271, 3.037)	0.410
Time	-0.855 (-3.286, 1.576)	0.479
Tiredness/Motivation	-0.521 (-2.566, 1.523)	0.607
Prefer Other Activities	-0.801 (-3.293, 1.692)	0.518
Diverse Interests	-1.969 (-4.344, 0.406)	0.101
Available Places to Walk	0.022 (-3.959, 4.002)	0.991
Neighborhood Safety	-5.366 (-12.097, 1.366)	0.114
Other Parent-Suggested Barriers	-3.246 (-6.339, -0.153)	0.040*

Results are from two separate linear regression models with outcome of parents' composite self-efficacy score for helping their child attain at least one hour of moderate intensity physical activity every day (scored from 4-20 where higher numbers indicate higher self-efficacy). Both models include adjustment for child body mass index (normal or underweight, overweight, obese), parent education level (some college or associate's degree vs. bachelor's degree or above), and family income ($100,000 or under vs. $100,001 or over). "Prefer other activities" refers to 'would rather participate in physical activities with your child other than walking.' "Diverse interests" refers to 'diverse interests between you and your child.' "Other parent-suggested barriers" included: child's lack of interest in walking outdoors (n=4, 8.0%), other children (n=2, 4.0%), child tiring out too quickly (n=1, 2.0%), and mindset (n=1, 2.0%). * indicates $p<0.05$.

Supplemental Table S2. Poisson regression results for examining associations with minutes per week that the child is physically active with their parent, after adjusting for child body mass index, parent education level, and family income.

	Rate Ratio (95% CI)	P-Value
Model 1: Number of barriers		
Number of barriers	0.882 (0.723, 1.075)	0.213
Model 2: Individual barriers		
Weather	1.006 (0.595, 1.702)	0.982
Time	1.112 (0.696, 1.776)	0.657
Tiredness/Motivation	1.020 (0.540, 1.926)	0.951
Prefer Other Activities	1.203 (0.638, 2.267)	0.568
Diverse Interests	0.422 (0.247, 0.721)	0.002*
Available Places to Walk	0.516 (0.200, 1.330)	0.171
Neighborhood Safety	0.916 (0.238, 3.521)	0.899
Other Parent-Suggested Barriers	0.264 (0.119, 0.590)	0.001*

Results are from two separate Poisson regression models with the outcome of minutes per week that the child is physically active with their parent. Both models include adjustment for child body mass index (normal or underweight, overweight, obese), parent education level (some college or associate's degree vs. bachelor's degree or above), and

family income ($100,000 or under vs. $100,001 or over). Rate ratios are exponentiated coefficients from the Poisson regression model, and the robust sandwich variance estimator was used to calculate confidence intervals and p-values. “Prefer other activities” refers to ‘would rather participate in physical activities with your child other than walking.’ “Diverse interests” refers to ‘diverse interests between you and your child.’ “Other parent-suggested barriers” included: child’s lack of interest in walking outdoors (n=4, 8.0%), other children (n=2, 4.0%), child tiring out too quickly (n=1, 2.0%), and mindset (n=1, 2.0%). * indicates p<0.05.
